# A Study of Nursing Progress and Its Development

**Published:** 1917-12-08

**Authors:** 


### THE MATRONS' AND SISTERS' DEPARTMENT.

### A Study of Nursing Progress and its Development.

THE question in the minds of all nurses at the present moment is, "What is the outlook for the College of Nursing?" To find an answer to it worthy of the important issues involved demands a modicum both of knowledge and of faith. It is very important to grasp the fact that the claims of the new College to the support of nurses do not rest alone on syllabuses and promises. They rest on achievements already won in the teeth of many difficulties, and on the greatness of aims already fulfilled to some extent and in certain quarters.

It is now pretty generally agreed that the right development of nursing in the near future calls first for the recognition of the probationer as a student, rather than as a poorly-paid assistant in hospital work; secondly, for the standardisation of the practical and theoretical teaching which forms the basis of all nursing education; thirdly, for the extension of opportunity to that higher grade of nurse whom Florence Nightingale had always in view. There are many other important points to be dealt with, but these three lie at the root of the matter. If they can be attained, the nursing profession will be immeasurably raised in status, and will be able to meet, with a spirit of confidence, such further developments as may lie in the future.

The measure of confidence to be extended to the College of Nursing must depend on the extent to which its promoters have shown their ability to recognise the need for these reforms and actually to aid in bringing them about. The present may be reckoned a transition period between nurse training as it was in the past, and nurse training as it can and ought to be in the future. Modern training-schools—London, provincial, Scotch, Irish —display every degree in the scale of quality, bad, indifferent, good, excellent. It must therefore be asked what earnest have nurses in general that, the promoters of the College have the power, as well as the will, to establish nurse training on sound and progressive lines? We have attempted during the last few months to bring before our readers the lines on which certain leading training-schools are conducted, to set forth the curriculum, the advantages offered to probationers under training, and any extended courses available for certificated nurses. Do nurses generally realise that an overwhelming majority of the matrons who are most successfully struggling to turn counsels of perfection into working possibilities, who are actually translating their ideals into practice, have now given their adhesion to the College, and are actively engaged in the task of framing its policy? We have all learned to distrust high-sounding syllabuses and programmes of reform, so fatally easy to compose, so hard of realisation, but when the life-work of certain leading spirits in the College of Nursing is studied, when, for instance, an effort is made to appreciate the conditions of nurse training at St. Thomas's Hospital, as recently set forth in these columns, it becomes evident that, there are among us women who have proved themselves capable of turning a bare scheme into a living reality. That is what the nursing world needs. This is what the College of Nursing can command.

Every step in nursing progress has been won by imagination and administrative skill, combined in the person of those whose position has enabled them to translate sound ideals into action. Outsiders may theorise and suggest, but it is to the matrons of the present day, and to the sisters who work with them in the training-schools, which lead the van of progress, that nurses must look to inaugurate the nursing of the future.

(*To be continued.*)

